# The challenges and opportunities of continuous data quality improvement for healthcare administration data

**DOI:** 10.1093/jamiaopen/ooae058

**Published:** 2024-08-01

**Authors:** Yili Zhang, Jennifer A Callaghan-Koru, Güneş Koru

**Affiliations:** Innovation Center for Biomedical Informatics, Georgetown University, Washington, DC 20007, United States; Department of Internal Medicine, University of Arkansas for Medical Sciences, Fayetteville, AR 72703, United States; Departments of Health Policy and Management & Biomedical Informatics, University of Arkansas for Medical Sciences, Fayetteville, AR 72703, United States

**Keywords:** data quality, data governance, healthcare administration, qualitative research, data quality improvement, Medicaid Management Information System

## Abstract

**Background:**

Various data quality issues have prevented healthcare administration data from being fully utilized when dealing with problems ranging from COVID-19 contact tracing to controlling healthcare costs.

**Objectives:**

(i) Describe the currently adopted approaches and practices for understanding and improving the quality of healthcare administration data. (ii) Explore the challenges and opportunities to achieve continuous quality improvement for such data.

**Materials and Methods:**

We used a qualitative approach to obtain rich contextual data through semi-structured interviews conducted at a state health agency regarding Medicaid claims and reimbursement data. We interviewed all data stewards knowledgeable about the data quality issues experienced at the agency. The qualitative data were analyzed using the Framework method.

**Results:**

Sixteen themes emerged from our analysis, collected under 4 categories: (i) Defect characteristics: Data defects showed variability, frequently remained obscure, and led to negative outcomes. Detecting and resolving them was often difficult, and the work required often exceeded the organizational boundaries. (ii) Current process and people issues: The agency adopted primarily ad-hoc, manual approaches to resolving data quality problems leading to work frustration. (iii) Challenges: Communication and lack of knowledge about legacy software systems and the data maintained in them constituted challenges, followed by different standards used by various organizations and vendors, and data verification difficulties. (iv) Opportunities: Training, tool support, and standardization of data definitions emerged as immediate opportunities to improve data quality.

**Conclusions:**

Our results can be useful to similar agencies on their journey toward becoming learning health organizations leveraging data assets effectively and efficiently.

## Introduction

### Background and significance

Healthcare administration organizations, such as state health agencies, have adopted various software systems to improve their effectiveness and efficiency in recent decades.^1^ Consequently, the volume of data maintained by these organizations, such as claims, payments and reimbursement, procedure, and provider data, has increased rapidly and substantially. Such data present tremendous opportunities to support day-to-day operations and decision-making as well as innovation and research in health services and public health.

However, various issues with data quality limit the benefits of healthcare administration data.^2^^,^^3^ Data quality is commonly defined as fitness for intended use, in that the data are complete, correct, and meaningful for a particular user’s goals.^4^ In general, data quality problems are prevalent in data residing in various healthcare information systems from medical registries,^5^^,^^6^ to healthcare assessment surveys,^7^ to electronic health records,^8–10^ to Medicaid Management Information Systems (MMIS).^11^ A lack of data quality was shown to present obstacles for smooth operations,^12^ results in financial losses,^13^ and contributes to medical errors.^14^ In a high-profile example in the United Kingdom, a single data error impacted the ability to perform contact tracing for a subset of the population in the early days of the COVID-19 pandemic.^15^ Approximately 16 000 positive tests were omitted from the official daily numbers, meaning that an estimated number of 50 000 potentially infectious people were not traced and told to self-isolate.

A data defect refers to a corrective change needed in data-lower defect counts are associated with higher data quality. An earlier study^11^ provides a comprehensive taxonomy of data defects encountered in healthcare administration data. In this taxonomy, data defects fall into 5 major categories, missingness, incorrectness, syntax violation, semantic violation, and duplication, and 17 subcategories. Preventing data defects and maintaining data quality is a timeless problem for organizations maintaining sizable healthcare data, due to multiple factors. First, the software systems that are used to collect and house data experience constant pressure to change and adapt to new requirements during their lifetimes.^16^ Alongside these changes, the programs, documentation, and data maintained in software systems steadily decay^17^^,^^18^ unless work is performed to improve quality as the system evolves.^19^ A second essential data quality challenge is that some data users can be separated from data producers in terms of organizational unit and time. This separation results in new or different data expectations, which should be addressed to improve data quality. Additionally, accidental reasons, such as inaccurate data entry, errors in system upgrades, and mistakes in data backups, can also negatively impact data quality.^11^

Given the multiple factors that work to erode data quality over time, *continuous data quality improvement*,^20^^,^^21^—that is, systematically identifying and resolving issues with data as an ongoing activity—becomes a critical need in today’s health organizations.^22^ Without adopting systematic approaches, managing data quality can become complex, difficult, and time-consuming. To date, a number of studies have focused on data quality assessment,^23–25^ providing data quality feedback,^26^^,^^27^ and performing data quality training for health system users.^28^ However, the processes that organizations routinely follow to manage and improve the quality of healthcare administration data are not well studied.

To address this gap, we conducted an in-depth investigation into the challenges and opportunities for continuous data quality improvement processes for healthcare administration data within a state health agency. State agencies maintain a large volume of data in disparate software systems, utilized across the organization for operational and analytical purposes. In an earlier study conducted in the same agency, our team identified substantial data quality problems, and nearly 3 million data defects in the Provider and Procedure subsystems of the MMIS.^11^ Overall, 9.74% of data cells in these 2 subsystems contained defects, pointing out substantial room for quality improvement. Understanding the existing data management processes, and opportunities and challenges for improvement, is essential to address this and similar data quality problems.

### Objectives

This research study had 2 objectives regarding healthcare administration data:

- Describing the currently-adopted approaches and practices for understanding and improving data quality, and
- exploring the challenges and opportunities to achieve continuous data quality improvement.

## Methods

We adopted a qualitative descriptive study approach^29^ to elicit rich contextual research data based on lived experiences and observations. We collected data through semi-structured interviews^30^ with data custodians and users at a state health agency (an institutional review board (IRB) approval was obtained from the University of Maryland, Baltimore County (Y17GK12046) prior to data collection), which establishes policies for the state’s Medicaid programs and manages them. MMIS, adopted in the late 1990s and revised in the 2000s, plays a central role in supporting daily operations and serving as a data source for analytics at the agency. MMIS data, the main healthcare administration data whose quality improvement was the subject topic for our interviews, contained data about Medicaid plans, eligibility, claims, reimbursements, providers, and procedures. In addition to supporting daily operations, MMIS data were analyzed for various decision-making purposes.

In this study, we adopted the expert sampling method.^31^ The phrase “data quality” did not appear in any position title or description at the agency. Through internal referrals, we collected the contact information of employees knowledgeable about data quality issues related to MMIS data. Due to their work responsibilities and activities, these individuals were known in the organization to be aware and concerned about the quality of data assets; therefore, they can generally be called “data stewards,” although the agency did not specifically designate or use such a title. The recruited participants had experience with the content of data maintained by the MMIS, and how it is managed, entered, reviewed, and utilized during which data quality issues were encountered. They also knew about past and current initiatives for improving data quality within the agency. Participation in the study was voluntary and involved no incentives.

The interview questions were based on a number of established systems analysis concepts and techniques,^32^ of which include problem analysis, activity duration analysis, activity-based costing, outcome analysis, and technology analysis. The questions were sent to participants 1 week ahead of the interview. The interviews were conducted remotely, recorded, and transcribed verbatim to ensure accuracy. Only 1 participant was interviewed in each interview. On average, each interview lasted for 1 h. The semi-structured nature of the interviews allowed for probing of participants with follow-up questions to develop a thorough understanding of participant’s experiences and opinions.^33^

We analyzed the data following the framework method.^34^ The analysis steps involved (1) data familiarization to identify emerging concepts, (2) constructing a conceptual framework with concepts and classifying them into categories, (3) coding/labeling data in transcripts according to the conceptual framework, (4) creating thematic charts to sort and synthesize the data under each concept. We developed simple scripts for labeling text, filtering text based on labels, and creating an index. We used Microsoft Excel to create thematic charts.

As typical in qualitative research, data analysis was an ongoing and iterative process, which started with the first interview. Two researchers independently performed coding. To ensure inter-rater reliability, Cohen’s Kappa^35^ analysis was performed, which involves identifying the number of agreements among the coders and dividing it by the total number of items. A score falling within the range of (81%–100%) is considered indicative of a high level of agreement.^36^ Any coding discrepancies were resolved through discussions and consensus. The analysis results continuously informed revisions to the interview guide to explore emerging themes. After preliminary analysis, we discussed themes and findings with agency officials following a member-checking approach. Their feedback informed the refinement of the coding framework and interpretation of themes.

## Results

We interviewed all reachable data stewards, a total of 12 participants, whose roles are shown in Table 1. Nine participants were health policy analysts, who reported using the data for making policy decisions. Six participants validated and corrected data as a part of their daily work. One participant was the supervisor of provider enrollment, and another one served as a claim adjuster who reviewed Medicaid billing and claim reports. Finally, 1 participant was a health IT business manager, who manages projects and access to the database.

**Table 1.** Participant roles and activities.

Participant ID^a^	Role	Activities related to data quality improvement
P1	Health Policy Analyst	Data validation and correction
P2	Supervisor of Provider Enrollment	Verify provider eligibility for Medicaid enrollment
P3	Medicaid Claim Adjuster	Review claim reports
P4	Health Policy Analyst	Data validation and correction
P5	Health Policy Analyst	Verify data for special healthcare services
P6	Health Policy Analyst	Data validation and correction
P7	Health Policy Analyst	Data validation and correction
P8	Health IT Business Manager	Manage IT projects and data resources
P9	Health Policy Analyst	Data validation and correction
P10	Health Policy Analyst	Data validation and correction
P11	Health Policy Analyst	Verify provider eligibility for Medicaid enrollment
P12	Health Policy Analyst	Data validation and correction

a Participants are listed from top to bottom with respect to the chronological order of interviews, P1 being the first participant interviewed.

### Conceptual framework

As we interviewed the participants, a number of interrelated concepts emerged with a Kappa score is 83.88%. These concepts were grouped under 4 categories in our framework as shown in Table 2: (i) Characteristics of data defects; (ii) Current processes and people issues; and (iii) Challenges and (iv) Opportunities in achieving continuous data quality improvement. These emerging concepts were repeated throughout the interview period as shown in Table 3 and selected representative quotes can be seen in Table 4.

**Table 2.** Conceptual framework.

Category	Concept	Description
(i) Characteristics of data defects	a. Variety… …… …… …… . .	A wide-range of data quality problems exist
	b. Obscurity… …… …… …. .	Data quality problems remain unknown and surface unexpectedly
	c. Difficulty… …… …… ……	It is often difficult to resolve problems
	d. Dependency… …… …… ..	Work on data defects exceeds organizational boundaries
	e. Severity… …… …… …… .	Leading to negative effects within and outside the agency
(ii) Current process and people issues	a. Ad-hoc approaches… …… .	Approaches to improve data quality varies based on the case
	b. Manual instruments… …….	Detect data quality manually with hard-copy documents
	c. Work frustration… …… … .	Participants are overwhelmed by redundant and manual operation
(iii) Challenges for continuous data quality imp.	a. Communication… …… …. .	Inefficient communication patterns while resolving defects
	b. Legacy systems… …… …. .	Antiquated systems used for operational reasons maintain data
	c. Lack of knowledge… …… . .	Users lack knowledge about systems, software, and data
	d. Different standards… …….	Different data standards are used by different stakeholders
	e. Data verification… …… … .	Access methods and authorization are needed to verify data
(iv) Opportunities for continuous data quality imp.	a. Standardization… …… …. .	Achieving a common understanding of data across the agency
	b. Tool support… …… …… . .	Adopting software solutions for data quality improvement
	c. Training… …… …… ……	Staff training on tasks associated with data quality improvement

**Table 3.** Concept saturation table (participants numbered w.r.t. chronological order of interviews).

	P1	P2	P3	P4	P5	P6	P7	P8	P9	P10	P11	P12
(i) Characteristic of data defects												
a. Variety … … … … … … … … … … … … … .	✓		✓		✓	✓	✓	✓	✓			
b. Obscurity … … … … … … … … … … … … .	✓		✓				✓	✓	✓	✓		
c. Difficulty … … … … … … … … … … … …. .			✓			✓	✓	✓	✓			✓
d. Dependency … … … … … … … … … … … .		✓		✓			✓	✓				
e. Severity … … … … … … … … … … … … …	✓	✓	✓			✓	✓	✓	✓	✓	✓	✓
(ii) Current process and people issues												
a. Ad-hoc approaches … … … … … … … … …			✓			✓	✓	✓	✓		✓	✓
b. Manual instruments… … … … … … … … …	✓	✓	✓	✓		✓	✓		✓	✓		
c. Work frustration … … … … … … … … … …	✓	✓	✓					✓	✓	✓		
(iii) Challenges for continuous data quality imp.												
a. Communication … … … … … … … … … … .	✓	✓	✓		✓	✓	✓	✓		✓	✓	
b. Legacy systems … … … … … … … … … … .		✓		✓	✓	✓	✓	✓				
c. Lack of knowledge … … … … … … … … … .	✓	✓	✓								✓	
d. Different standards … … … … … … … … …	✓	✓		✓	✓				✓		✓	✓
e. Data verification … … … … … … … … … …		✓	✓		✓	✓	✓		✓	✓	✓	✓
(iv) Opportunities for continuous data quality imp.												
a. Standardization … … … … … … … … … … .	✓		✓	✓						✓	✓	✓
b. Tool support … … … … … … … … … … … .	✓	✓	✓	✓	✓		✓		✓			
c. Training … … … … … … … … … … … … …	✓	✓	✓	✓	✓	✓					✓	✓

**Table 4.** Selected representative quotes from the interviews.

Q1. “Sometimes when those provider numbers get reported to us, they’re missing or with leading zeros. Sometimes they report the base number but not the suffix. Sometimes there are typos.” (P9) Q2. “I realize there are problems when I actually need to use the data to answer some of questions I need to answer.” (P10) Q3. “The thing is when we might be looking for one problem, we’ll notice an additional one. It’s like you have one tree branch, branching off again. What it just affected keeps getting bigger and bigger and bigger. You find out that there’s more than one aspect you thought was wrong. There could be a lot.” (P3) Q4. “When there was no consensus with some data uniformity, we actually had to kind of go around and like have a few extra meetings with the programming folks who fully understand how some sets of data are formulated.” (P8) Q5. “…, the problems were often brought to my attention by either people in other departments or the provider that we’re working with who noticed an issue.” (P8) Q6. “Sometimes it’s not a matter of just correcting the data in the system. Sometimes it actually requires another party to actually reach out to the provider to either verify information that is not correct, or engages that needs to be made, or other information.” (P3) Q7. “If the provider file that (claims)… The quality of data is not there. And that will affect billing. It will affect claims. It will affect a mess.” (P3) Q8. “… We get a report that says a provider is doing a hundred services in one day then that seems unrealistic then there could be consequences on that end too. So it’s important to understand and make sure that our data is accurate…” (P1) Q9. “I mean each time it’s different about (data quality) control. I mean especially when you don’t have a standard process or procedure for going through it.” (P6) Q10. “You know if I would ever switch jobs or anything like that, I don’t know whether the next person knows the fixes I have done or what to look for.” (P12) Q11. “I mean if you have bad data you might have to go through certain data sets that could be tens of thousands of lines… might have to go through them line by line to sort out a field.” (P9) Q12. “You know, right now we are still very very much stuck in using hard copies and everything in papers, where we haven’t gone completely electronic by a long shot and you know identifying that problems and changing it by papers on an individual basis that takes a lot of time and energy.” (P2) Q13. “So each time we receive a form on a request, I need to manually type their name again, maybe just like for record. Everything needs to keep record. I think that’s kinda waste of time.” (P10) Q14. “I had to go through several hundred lines of these reported services. And to determine which ones were clear enough that we should pay for which ones were ambiguous enough that we should not pay for and take it back to the hospital.” (P9) Q15. “You know I commonly ask for reports that I believe are clear but when I get the data it doesn’t answer my question.” (P1) Q16. “I think the one biggest issues is that I look at my cabinet right now, and I can count one, two, three, four, five, six, or seven eight different like you know, two inches binders just filled with these really odd printouts of how MMIS to works.” (P4) Q17. “If our system cannot do that task, because the system is so old. They need to turn this like certain part of striking process. They need to turn in to a third party. Yeah, I think maybe that would be the most costly part.” (P7) Q18. “I still have a lot of learning to do. If you were asking somebody who are more familiar with it (MMIS), they would have system ways to do it (data quality improvement).” (P11) Q19. “Usually the key error is lack of understanding or knowledge of what fields are relevant depending upon which provider criteria.” (P3) Q20. “For instance, we have multiple systems from our MMIS system which captures all Medicaid eligibility information and provider information and billing information to our system, which captures a lot of service information like program eligibility information, medical, clinical information. Comparing that to federal data from CMS, their data and their availability of data with census data etc My biggest problems come from having five ten different sources and pull them all together and make sense out of more of a macro trend.” (P5) Q21. “And you know CMS could say ‘hey we’re checking that you’re incompliance with these rule’. The rules that we’ve never had to be compliance with before. And um you know it takes a long time to update our system to get into compliance with them.” (P12) Q22. “To use specific data to determine whether or not the claim were paid twice or not, you know, that specific information sometimes lost. Because it wasn’t there or we weren’t able to require that.” (P2) Q23. “… Sometimes I just don’t really capture the full picture of you know what’s going on beyond this building, all you have is the data that you have on your side.” (P8) Q24. “The way that you collect that data, and also define what data actually means is so important. You know, a piece of data as far as what it means to us doesn’t necessarily mean the same thing to the provider.” (P10) Q25. “And I wish there would be a more prompt thing like how would this report be used and what do you want the end product to look like something like that. Or what do you expect to the result to look like, just a little bit more information I think kinda help.” (P1) Q26. “… why it takes me so long … if I had more experience in the software and automated data quality checks then I think it wouldn’t be such a loss but because I have to manually look up everything that’s challenging.” (P1) Q27. “And if they’re missing information that is basically a retraining issue. It’s uh you know sit down say ‘Okay, well, this is why, this is wrong, this is what it needs to be.’ So, it’s from a training perspective where the data quality seems to bleed.” (P3)

#### (i) Characteristics of data defects

- ***Variety.*** Six participants mentioned observing a wide variety of data defects, that is, corrective changes needed in data, which included mismatches, inconsistencies, missingness, and incorrect data across different categories. For instance, mismatches in National Provider Identifiers (NPIs) led to confusion during Medicaid claim processing, while inconsistent patient activation statuses caused delays in filing claims and receiving reimbursements (see Q1 in Table 4). Another recurrent concern is incorrect data caused by typing errors, delayed data updates, or data transportation. Such incorrect data increased the risk of errors during the payment of claims.
- ***Obscurity.*** Six participants reported that data defects were obscure, and they were often discovered during the actual use of data (Q2). Therefore, it is uncertain when and how the data quality issues will be discovered. For instance, inconsistencies across data records were frequently identified during data analysis, leading to further investigations that revealed additional underlying problems that were previously unknown (Q3).
- ***Difficulty.*** One-half of the participants reported experiencing difficulties in resolving the identified issues. One participant described reaching out to relevant individuals to seek guidance and find a conclusive answer (Q4). Inadequate or missing documentation was often encountered during this process. Sometimes, the individuals who could possess the knowledge about data no longer worked at the department.
- ***Dependency*.** Participants mentioned numerous data dependencies requiring defect detection and resolution that exceeded organizational boundaries. For instance, two participants reported that they learned about certain data quality problems only after receiving complaints from healthcare providers regarding claim denials and billing discrepancies (Q5). Subsequently, the participants needed providers’ collaboration to resolve data quality problems. In addition, one participant noted that the data collected through IT software systems (eg, attestation data collected from providers about their Medicaid eligibility) had many problems that required investigation by the agency, which resulted in an outreach to the IT vendor to fix the issues (Q6).
- ***Severity*.** Ten participants provided specific examples of severe outcomes, such as communication and operational inefficiencies, caused by data defects (Q7). Also, the participants mentioned that data quality improvement is critical for preventing fraudulent activities and financial losses. For example, an unlikely number of services detected in data raises a red flag for potential fraud; however, data quality must be trusted to act on such signals (Q8).

#### (ii) Current process and people issues

- ***Ad-hoc approaches*.** Seven participants reported relying on convenient, improvised, and varying solutions to improve data quality (Q9). When skilled personnel leave the agency, the prior solutions may no longer be useful because they are simply not retained (Q10).
- ***Manual instruments*.** Eight participants stated that they manually track, review, and detect data quality problems. Data stewards often review data by eyeballing it on a monitor line-by-line to verify the data (Q11). When they have to switch between paper documents and the computer to confirm information, it becomes time-consuming and fatiguing (Q12). In some circumstances, this process repeats several times because participants do not find the problem reported. Many documents in their workflows were still paper-based, such as the data request forms (Q13) and the rule matrix that defines business rules, for example, valid services for each provider type.
- ***Work frustration*.** Six participants expressed frustration about the inadequacy of the ad-hoc solutions they were using for data quality control, which burdens participants with non-uniform and mostly manual efforts to comprehend, track, and resolve data quality problems. One participant mentioned having to go through hundreds of lines of reports in order to make a payment decision (Q14). For one participant, it became frustrating to come across additional data defects while trying to remove the initial ones (Q3).

#### (iii) Challenges

- ***Communication*.** Nine participants brought up inefficient communication patterns while resolving defects within the agency (Q15). Furthermore, communication with external parties like providers, clinicians, or patients can be difficult and prolonged (Q6). Participants mentioned using limited information to explain data quality problems to external stakeholders, which can lead to miscommunication as these stakeholders may not always provide an accurate answer.
- ***Legacy systems*.** When dealing with data quality problems, data stewards are expected to learn the legacy systems used for operational purposes, such as MMIS, from several binders of manual documents, which makes it challenging for them to fully comprehend the information and efficiently use it (Q16). In addition, the agency contracts with IT vendors to build satellite systems that provide data to, or consume data from, the legacy systems (Q17). As system dependencies increase, detecting and resolving data quality problems requires communication with external parties.
- ***Lack of knowledge*.** Four participants identified the lack of knowledge regarding software systems, data, and data quality requirements as a significant challenge for data quality improvement. The absence of systematic training on the system, apart from paper-based manuals, leads to many individuals in the department being unfamiliar with the system, except for a handful of people; staff with limited knowledge introduce data errors while using the system (Q18, Q19).
- ***Different standards*.** Seven participants expressed difficulties in communicating medical indexes and codes between MMIS and other systems, especially when working on projects that require data from diverse stakeholders. They also highlighted the challenge of composing consistent reports due to different data requirements from Federal and State organizations (Q20). Data requirements also changed over time. For example, the Center for Medicare & Medicaid Services (CMS) published new rules that changed Medicaid data format that was not compliant with the MMIS system (Q21).
- ***Data verification*.** Data stewards did not always have access to data they needed to verify in the agency (Q22), often necessitating time-consuming communications with people at other organizations (Q23). In addition, data in different information systems were not synced with each other in a timely manner.

#### (iv) Opportunities

- S***tandardization***. Five participants recommended using a data catalog to standardize data definitions within the agency (Q24). When definitions were not commonly understood, errors occurred due to insufficient and different understanding of data and how it should be used. One participant suggested that reporting standards could facilitate data quality improvement (Q25).
- ***Tool support.*** One-half of the participants recommended building supports into tools to automate tedious and repetitive tasks, such as applying certain constraints expressed as queries or statements to detect data defects (Q26). The information reported by supportive tools must be easy to understand. The participants also mentioned that they desired tools for dispatching and tracking the tasks needed for data quality improvement.
- ***Training.*** Eight participants considered training about the system, data, and data quality to be necessary for data quality improvement. According to one participant, training on the criteria used for data quality assessment could lead to better data quality reports (Q27).

## Discussion

Our study revealed that a state health agency encountered diverse challenges with data quality and its improvement. The agency primarily relied on ad-hoc and manual processes to improve data quality, and there was generally a lack of documentation of data and processes. Data quality control processes can be considered on a spectrum of maturity, a model commonly adopted to characterize the “extent to which a process is explicitly defined, managed, measured, controlled, and effective.”^37^ When considered against a data-quality maturity model, which consists of various levels describing the sophistication and effectiveness of data quality management practices, the characteristics observed in the organization are consistent with the initial level, characterized by chaotic, ad-hoc and manual processes.^37^

The agency was mostly reactive in dealing with data quality issues, which were typically discovered when data were needed or used. A reactive approach cannot effectively prevent defect proliferation, also called the Butterfly Effect,^38^ which contributes to the likelihood and severity of negative outcomes associated with using bad data.^39^ One proactive approach is to try to ensure data quality at the time of data collection. For this purpose, developing better system documentation and providing training to end-users to develop skills for using systems and data^40^^,^^41^ can be useful.

Agency staff desired to replace ad-hoc and manual data quality improvement solutions with best practices such as systematic, sufficiently documented, and automated solutions that support communication and teamwork. This finding is consistent with earlier studies demonstrating that manual data operations are labor-intensive^42^ and are more susceptible to error^43^ in contrast to electronic and automated operations. A mechanism for computer-supported data verification is not only necessary for data quality improvement but also vital for daily healthcare administration operations such as reimbursing claims.

To support data quality improvement, similar agencies can benefit from maintaining a macro view of the data standards applicable to various data sources, and adopt IT solutions following data standards as much as possible.^10^^,^^44^ Adoption of data standards (eg, common data models^45^ and HL7^46^) is crucial for facilitating data sharing within and across institutions,^47^ and checking for data errors, thus, contributing to data quality. Furthermore, within the organization, differences in data definitions and usage associated with business rules require additional attention for achieving organizational consensus on data standards and definitions. For this purpose, developing and using a data catalog can lead to a common understanding of the data elements at state agencies with data challenges. Preferably, such a data catalog should support the versioning of data definitions to provide the historical context needed to understand past decisions. Earlier studies suggest that proper documentation and usage guidelines are critical for data quality improvement.^48^

The participants also associated the use of legacy software systems, such as MMIS, with various reasons contributing to lower data quality, including interfaces and features that are hard to modify. These observations are consistent with earlier research which identified legacy systems as a significant contributor to data quality issues.^49^ For example, MMIS lacked effective automated error identification functions, making it difficult for users to prevent data entry errors and detect data defects emerging during various operations.

Over time, unless work is performed to prevent it, systems tend to become legacy systems due to changes in technology and requirements. Therefore, it is important for the state and federal agencies overseeing healthcare administration to budget for the modernization of healthcare administration systems aiming to restructure or replace legacy systems. Consequently, the structural quality of healthcare administration systems and the quality of data maintained in those systems can be improved. Modernization of large-scale legacy systems is not a straightforward problem; there are a number of well-publicized troubled^50^ and canceled^51^ modernization projects, resulting in a loss of resources. Moving into the future, IT procurements of cloud-based software-as-a-service solutions will likely push software modernization efforts increasingly to the vendor side. Therefore, working with reputable and successful vendors and maintaining productive relationships with them will be important for software modernization. Software upgrades also require caution to avoid data damage and distortions. However, with enough attention, those problems can be easier to avoid and manage compared to the legacy-system problems frequently mentioned by our participants.

Similar to other empirical studies, our study has certain limitations. The participants were recruited from a single state health agency, which funded the study. Our sample size was small but consisted of relevant and knowledgeable participants. Although we believe we identified and interviewed all participants knowledgeable in data quality improvement in the agency, it is possible that some individuals with relevant experiences were not identified. The study context was mainly determined by the collection and use of MMIS data for operational and analysis purposes in this agency. While the rich contextual results we obtained shed light on the obstacles to data quality improvement in this agency, it should be noted that the issues and their severity may be different in other agencies. Future quantitative studies investigating similar research questions in a larger sample of agencies can lead to potentially generalizable results.

## Conclusion

This qualitative case study explored the pressing challenges and opportunities of continuous data quality improvement faced by the data stewards in one state health agency. Our results provide empirically-based recommendations, which can be interpreted and used by similar agencies in their initiatives to improve the quality of healthcare administration data.

A key observation we have made is that data quality improvement is a socio-technical problem involving multiple stakeholders, not solely a technical problem. The organizational context of an agency, determined by its healthcare policies, business rules, software systems in use, and organizational culture, needs to be carefully considered to achieve continuous data quality improvement. Within that context, data quality improvement should be planned and implemented as a set of ongoing processes, rather than a one-time project with specific start and end dates. At the same time, state health agencies should continuously comply with the external rules impacting how they should store and manage data (eg, CMS rules) and mitigate the impact of external data quality issues such as those caused by the healthcare providers and system-level processes.

Future studies can focus on modeling collaborative workflows responding to the right challenges and opportunities of data quality improvement in specific agencies. An accumulation of results can lead to a set of empirically-based model workflows and accompanying software tools that can be customized to maximize effectiveness and efficiency in continuous data quality improvement.

## Data Availability

The qualitative data collected in the study cannot be shared for ethical and privacy reasons and due to the IRB restrictions.
